# Simultaneous HPTLC Determination of Rabeprazole and Itopride Hydrochloride From Their Combined Dosage Form

**DOI:** 10.4103/0250-474X.43004

**Published:** 2008

**Authors:** A. Suganthi, Sofiya John, T. K. Ravi

**Affiliations:** Department of Pharmaceutical Analysis, College of Pharmacy, Sri Ramakrishna Institute of Paramedical Sciences, Coimbatore-641 044, India

**Keywords:** Simultaneous estimation, HPTLC, rabeprazole and itopride

## Abstract

A simple, precise, sensitive, rapid and reproducible HPTLC method for the simultaneous estimation of the rabeprazole and itopride hydrochloride in tablets was developed and validated. This method involves separation of the components by TLC on precoated silica gel G60F254 plate with solvent system of n-butanol, toluene and ammonia (8.5:0.5:1 v/v/v) and detection was carried out densitometrically using a UV detector at 288 nm in absorbance mode. This system was found to give compact spots for rabeprazole (Rf value of 0.23 0.02) and for itopride hydrochloride (Rf value of 0.75±0.02). Linearity was found to be in the range of 40-200 ng/spot and 300-1500 ng/spot for rabeprazole and itopride hydrochloride. The limit of detection and limit of quantification for rabeprazole were 10 and 20 ng/spot and for itopride hydrochloride were 50 and 100 ng/spot, respectively. The method was found to be beneficial for the routine analysis of combined dosage form.

Rabeprazole and itopride hydrochloride^1^ are available in combined capsule dosage form for the management of gastro esophageal reflux disease (GERD). Literature survey reveals that various analytical methods^2^–^9^ have been reported for rabeprazole and itopride hydrochloride in single dosage forms. The present paper aims to report a simple HPTLC method for estimation of rabeprazole and itopride hydrochloride in their combined dosage form.

Rabium-plus containing 20 mg/tab of rabeprazole and 150 mg/tab of itopride hydrochloride, which are manufactured and marketed by Intas Ltd. was estimated. Reference standard of rabeprazole and itopride hydrochloride were kindly supplied as a gift sample by Grandix Pharmaceuticals, Chennai and Abbott Laboratories, Goa. All chemicals and reagents used were of analytical grade and were purchased from SD Fine Chemicals, Mumbai. A Camag HPTLC system comprising of Camag Linomat V semi automatic sample applicator, Hamilton Syringe, Camag Twin trough chamber, Camag TLC Scanner-3, Camag WinCATS Software and stationary phase precoated silica gel G60 F_254_ were used.

The TLC plates were pre-washed with methanol and activated by keeping at 115° for about 30 min. The samples were spotted in the form of bands of width 6 mm with Hamilton microlitre syringe on the precoated silica gel G_60_F_254_ plate (10×10 cm), slit dimension was kept at 5×0.45 mm, respectively. The mobile phase used was n-butanol:toluene:ammonia (8.5:0.5:1 v/v/v), chamber and plate saturation time of 30 min, migration distance allowed was 85 mm, linear ascending development was carried out in 10×10 cm, twin trough glass chamber. Subsequent to the development TLC plates were dried in a current of air. Densitometric scanning was performed at 288 nm. The source of radiation utilized was deuterium lamp emitting a continuous UV spectrum.

Standard stock solution mixture of rabeprazole and itopride hydrochloride were prepared in methanol of 200 μg/ml and 1500 μg/ml. Aliquots of standard solution having concentration ranging from 40-200 ng/spot and 300-1500 ng/spot (0.2, 0.4, 0.6, 0.8 and 1.0 μ1) of rabeprazole and itopride hydrochloride were applied on the TLC plate. The TLC plate was dried, developed and analysed as described earlier.

Twenty tablets of two different batches were chosen, weighed, powdered, transferred into a volumetric flask and extracted with methanol. The extract was filtered through Whatmann filter paper No 41 and residue was washed with methanol. Aliquots of 0.8 μl (160 ng/spot of rabeprazole and 1200 ng/spot of itopride hydrochloride) were applied on the precoated silica gel G60 F_254_ TLC plate. From the peak area, the amount of rabeprazole and itopride hydrochloride in formulation was simultaneously calculated using the respective calibration graph. The amount obtained per tablet and percentage label claim are shown in Table 1.

**TABLE 1:** RESULTS OF ANALYSIS OF FORMULATION

Drug	Amount (mg/tablet)		% label
		
	Labelled	Estimated	claim±SD*
Rabeprazole	20	19.60	98.41±0.1871
Itopride HCl	150	149.91	99.94±0.4557

* Mean±standard deviation of six determinations

The method was validated for linearity, accuracy, limit of detection, limit of quantification, inter-day and intra-day assay precision, repeatability of measurement and repeatability of sample application. Samples were applied, the plate was developed with the mobile phase and the peak areas were noted. The mobile phase consisted n-butanol, toluene and ammonia (8.5:0.5:1 v/v/v), gave R_f_ values of 0.23 for rabeprazole and 0.75 for itopride hydrochloride. A good linear relationship was obtained over the concentration range of 40-200 ng/spot of rabeprazole and 300-1500 ng/spot of itopride hydrochloride, respectively. The linear regression data showed a regression co-efficient of 0.99848 for rabeprazole and 0.99030 for itopride hydrochloride. The repeatability of sample application and measurement of peak area were expressed in terms of %RSD for n=3 observation. The study revealed intra and inter day variation of rabeprazole and itopride hydrochloride are expressed in percentage RSD as shown in Table 1.

The LOD with signal/ noise ratio were found to be 10 and 50 ng/spot for rabeprazole and itopride hydrochloride respectively. The LOQ with signal/ noise ratio was found to be 20 and 100 ng/spot for rabeprazole and itopride hydrochloride, respectively. Intra-day assay precision was found by analysis of standard drug at three times on the same day. Inter-day assay precision was carried out using the standard drug at three different days, and percentage relative standard deviation (% RSD) was calculated. The RSD was found to be less than 2 for both inter-day and intra-day assay precision. Repeatability of sample application was assessed by spotting 0.8 μl of drug solution, 6 times. From the peak areas, the percentage RSD was determined.

Repeatability of measurement was determined by spotting 0.8 μl of standard drug solution on TLC plate, after development, spot was scanned six times without changing its position. The percentage RSD was found to be within the limits. The recovery study was carried out at two levels, 50% and 100%. The complete validation parameters are shown in Table 2.

**TABLE 2:** METHOD VALIDATION PARAMETERS

Parameters	Rabeprazole	Itopride hydrochloride
LOD (ng/spot)	10	50
LOQ (ng/spot)	20	100
Linearity(ng/spot)	40-200	300-1500
Correlation coefficient	0.99848	0.99030
% Recovery studies		
50%±SD	101.04±0.41	100.61±0.52
100%±SD	100.80±0.37	99.19±0.24
Precision		
Intraday (%RSD)	0.7010	0.3373
Interday (%RSD)		
1^st^ day	0.8247	0.8748
2^nd^ day	1.2803	1.3287
Repeatability of application	0.2494	0.2702
Repeatability of measurement	0.7510	0.3505

SD= Mean±Standard deviation of six determinations. Recovery study was performed for one formulation only. % RSD= Percentage relative standard deviation.

Hence the developed HPTLC technique is simple, precise, specific and accurate, statistical analysis proved that the method is repeatable and selective for simultaneous analysis of rabeprazole and itopride hydrochloride as bulk drugs and in pharmaceutical dosage forms without any interference from the excipients.
